# E2-2, a novel immunohistochemical marker for both human and monkey plasmacytoid dendritic cells

**DOI:** 10.1007/s41048-016-0023-6

**Published:** 2016-04-11

**Authors:** Jianping Ma, Haisheng Yu, Xiangyun Yin, Menglan Cheng, Quanxing Shi, Zhao Yin, Xiaohua Nie, Wang Shouli, Liguo Zhang

**Affiliations:** Key Laboratory of Infection and Immunity, Institute of Biophysics, Chinese Academy of Sciences, Beijing, 100101 China; University of Chinese Academy of Sciences, Beijing, 100080 China; Department of Cardiology, 306th Hospital of PLA, Beijing, 100101 China

**Keywords:** pDC, E2-2, Monoclonal antibody, Immunohistochemistry

## Abstract

Plasmacytoid dendritic cells (pDCs) play important roles in initiating and regulating immune responses. pDC infiltration has been documented in multiple pathological lesions including infections, tumors, and autoimmune diseases, and the severity of pDC infiltration correlates with disease progression. However, a specific antibody for identifying pDCs by immunohistochemical staining on paraffin-embedded tissue sections is still lacking. Here, we developed a novel antibody targeted E2-2, a transcription factor preferentially expressed in pDCs. The antibody stains the nuclei of pDCs specifically in immunohistochemical analysis of various tissues from both human and rhesus monkey. This novel antibody will serve as a beneficial tool for pDC-related basic research and clinical investigation.

## INTRODUCTION

Plasmacytoid dendritic cells (pDCs), which are rare but crucial component of the immune system, serve as a bridge linking innate and adaptive immunity (Liu 2005). pDCs can produce 100–1000 times more type I interferon (IFN-I) than any other cell types upon viral infections or other stimulations (Siegal et al. 1999; Liu 2005; Reizis et al. 2011; Swiecki and Colonna 2015). In the recent years, the involvements of pDCs in multiple diseases, such as chronic viral infections, autoimmune diseases, and different types of cancers, have been extensively investigated (Tang et al. 2010; Vermi et al. 2011; Swiecki and Colonna 2015). pDC infiltration in the lesion area has been reported in the aforementioned diseases and the severity of pDC infiltration correlates with disease progression. In HIV/SIV infections, for example, pDCs migrate to lymphoid organs or mucosal tissues and contribute to pathological changes in these areas (Nascimbeni et al. 2009; Kwa et al. 2011; Li et al. 2013). Infiltration of activated pDCs to skin lesions was also reported in psoriasis, and pDCs may promote the morbidity through IFN-I dependent mechanism (Nestle et al. 2005; Reizis et al. 2011). Besides, in human tumors such as ovarian cancer, pDCs were found to be accumulated in the tumor sites through upregulation of several chemokine receptors (Zou et al. 2001; Vermi et al. 2011). The infiltrated pDCs may contribute to the induction and maintenance of immunotolerance in the tumor microenvironment (Vermi et al. 2011). The identification of immune cells by immunohistochemical analysis is essential for both clinical investigation and basic research. However, a specific antibody for pDC staining in paraffin-embedded tissue sections is still lacking.

E2-2, which is also called transcription factor 4 (TCF4), immunoglobulin transcription factor 2(ITF2), or SL3-3 enhancer factor 2 (SEF2), belongs to a family of basic helix-loop-helix transcription factors named E proteins. E2-2 is highly and preferentially expressed in both human and murine pDCs (Cisse et al. 2008). E2-2 binds the promoters and enhancers with E-box sequence and controls a series of genes that are critical for pDC development and maintenance (Cisse et al. 2008; Forrest et al. 2014; Cheng et al. 2015). In this study, we developed an anti-E2-2 monoclonal antibody, which could specifically bind to the nucleus of pDCs on paraffin-embedded tissue sections. This novel antibody will serve as a beneficial tool for pDC-related basic research and clinical investigation.

## RESULTS

### E2-2 is specifically expressed in pDCs

**Fig. 1:**
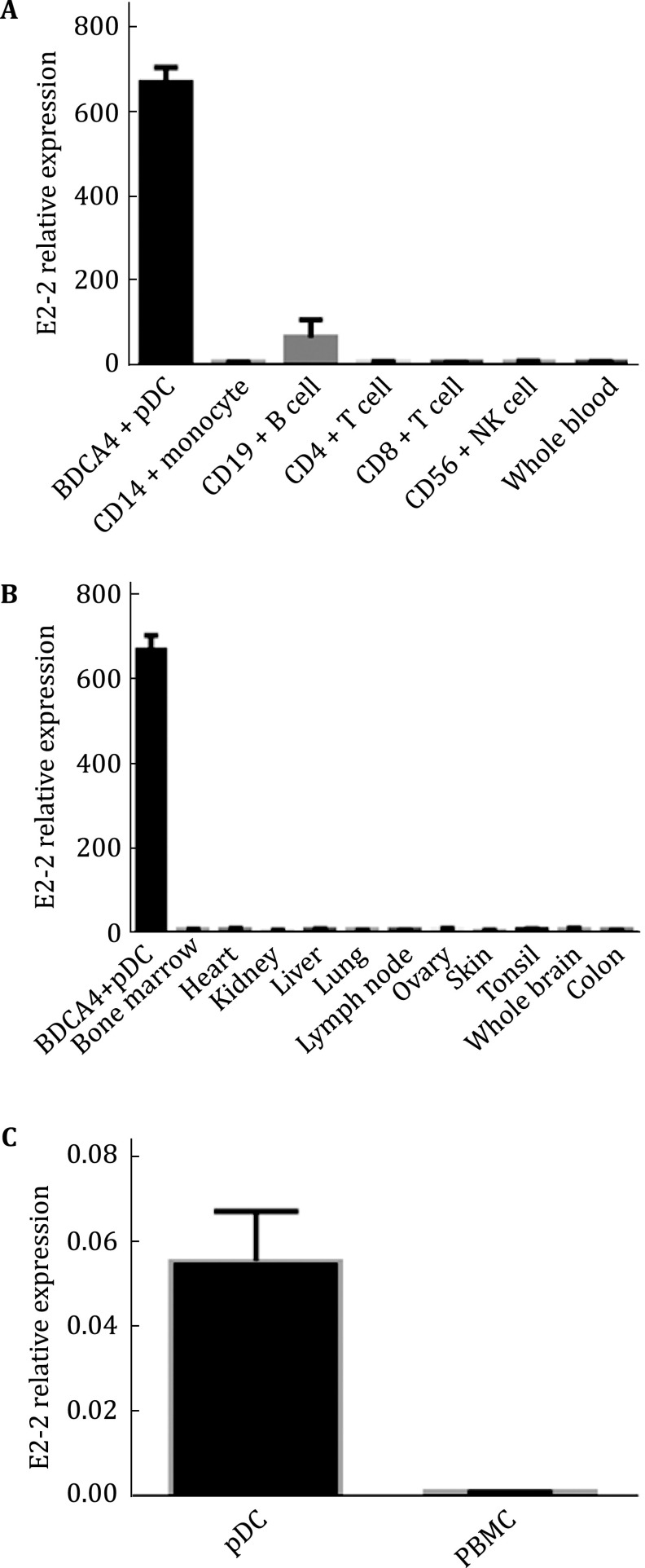
E2-2 expression profile in different immune cells and different tissues. **A** Comparison of E2-2 expression level in pDC and other immune cells in human blood. **B** Comparison of E2-2 expression level in pDC and tissues from different human organs. (Data from BioGPS database, http://biogps.org/#goto=genereport&id=6925.) **C** The relative mRNA level of E2-2 in pDC and total peripheral blood mononuclear cells by RT-PCR. (Data were based on three independent experiments.) The data are presented as mean ± SEM of results from two samples in BioGPS cDNA array tests and three different samples in the real-time PCR test

We compared the expression of E2-2 in different immune cells and tissues with data retrieved from BioGPS, an online open database of cDNA array (http://biogps.org/#goto=genereport&id=6925). E2-2 is highly expressed in pDCs, whereas its expression in T lymphocytes (T cells), natural killer cells (NK cells), and monocytes are at a basal level. Although B lymphocytes (B cells) also express E2-2, the RNA level is much lower than that of the pDCs (Fig. 1A). In addition, there is no E2-2 expression in non-lymphoid tissues, including bone marrow, heart, kidney, liver (Fig. 1B). E2-2 mRNA level is also low in lymphoid tissues such as tonsil and lymph nodes. This may be due to the rarity of pDC in lymphoid tissues. We also confirmed the result with real-time PCR and found E2-2 was highly expressed in enriched pDCs while total peripheral blood mononuclear cells (PBMC) showed nearly no expression (Fig. 1C). In summary, the transcription factor E2-2 is highly and preferentially expressed in pDCs and could serve as a pDC-specific marker.

### The stable secondary structure exists in the latter half of the E2-2 protein

**Fig. 2:**
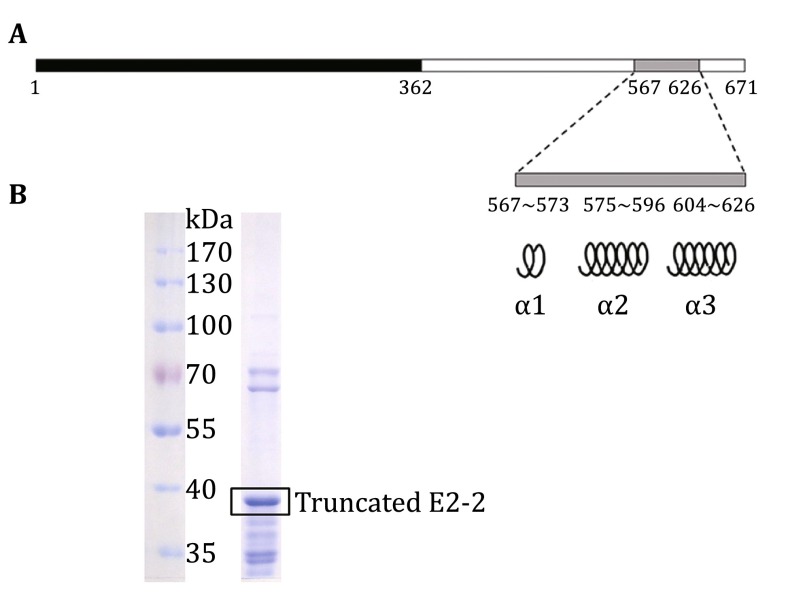
The expression of truncated E2-2 as immunogen. **A** The secondary structure of E2-2 protein was analyzed with Jpred4 as described in the method section. One region with three alpha-helix structures (amino acid sequence from 567 to 626) was shown. The second half (amino acid sequence from 362 to 671) was selected as the immunogen. **B** The purified E2-2 polypeptide was analyzed in SDS-PAGE gels and stained with Coomassie Brilliant Blue. The band between 35 and 40 kDa was truncated E2-2

**Fig. 3:**
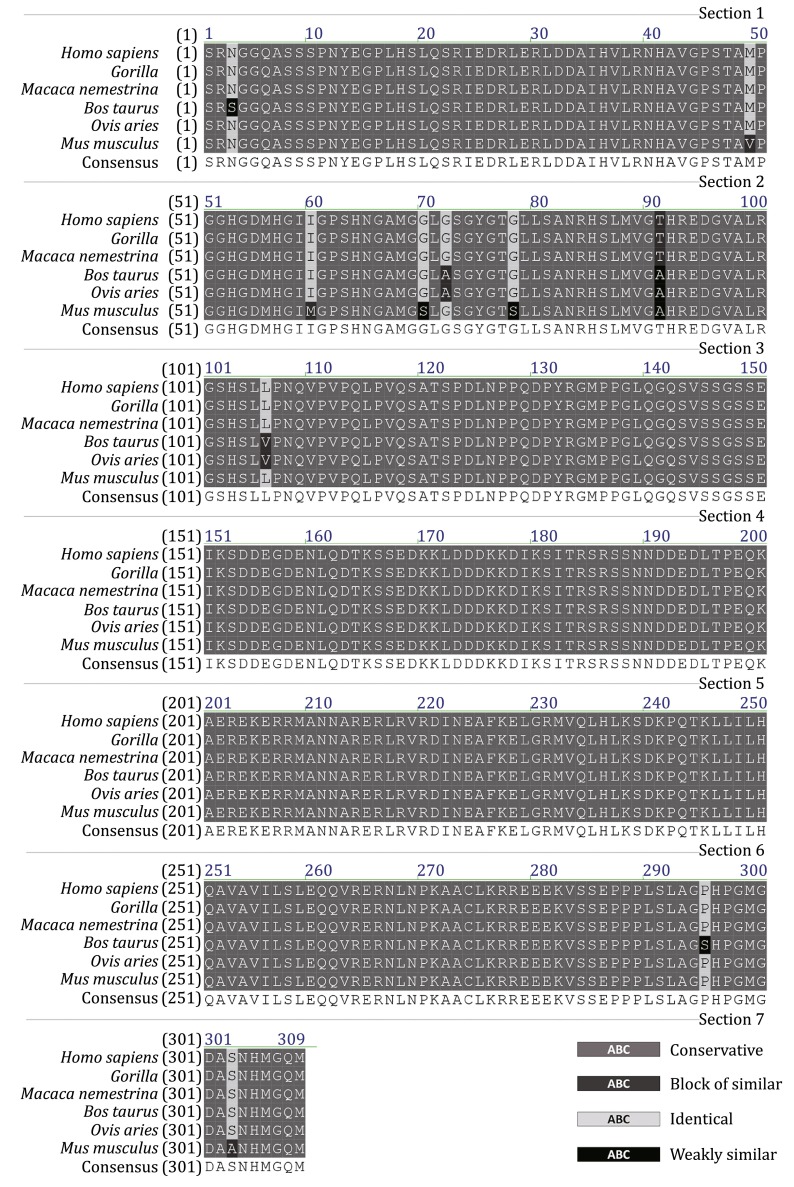
The alignment of E2-2 sequences between different species

As E2-2 is preferentially expressed in pDCs, we chose this molecule as the target for the pDC immunohistochemical staining. For subsequent monoclonal antibody development, it is crucial to prepare synthetic E2-2 protein/peptide with stable structure to be used as immunogen. We analyzed amino acid sequence with the online protein secondary structure prediction server-Jpred 4 and the result showed only three short alpha-helix domains existing in the latter half of the protein, with the remaining sequence having no definite structures predicted by the server (Fig. 2A). In addition, previous study also demonstrated that there are multiple alternative splicing sites in the first half of E2-2 (Sepp et al. 2011). As a result, we selected and cloned the latter half of the E2-2 protein (amino acid sequence from 362 to 671) for in vitro expression (Fig. 2B). In addition, results of amino acid sequence alignment among several species of mammals indicate that the selected fragment is conserved, and antibody targeted to this fragment may cross-react with other species as well (Fig. 3).

### The novel monoclonal antibody specifically binds E2-2

**Fig. 4:**
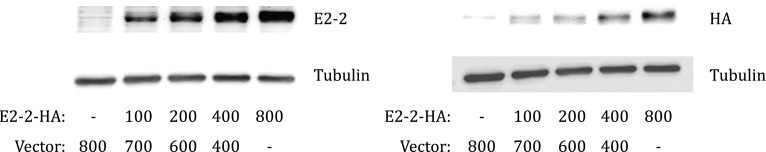
The binding specificity of 10F7 to E2-2 by Western blot. The binding of 10F7 was tested with HEK293T cells transfected with different quantities (ng/well) of E2-2 expressing plasmid. The polyclonal antibody was used to stain the HA-tagged E2-2 protein as well. The tubulin was used as an internal loading control

After 4 times of immunization with the purified E2-2 fragment mentioned above and screening based on enzyme-linked immunosorbent assay (ELISA), we selected one clone (10F7), which showed highest binding ability during the screening, and tested its performance in the subsequent experiments. We found that the clone 10F7 could bind E2-2 specifically in Western blot, as the band density increased as the escalated dosage of E2-2 expression plasmid transfected in the HEK293T cells (Fig. 4, lane 2–5), whereas non-transfected HEK293T cells showed no E2-2 expression (Fig. 4, lane 1).

### The 10F7 antibody can be used to stain human pDCs on paraffin-embedded tissue sections

**Fig. 5:**
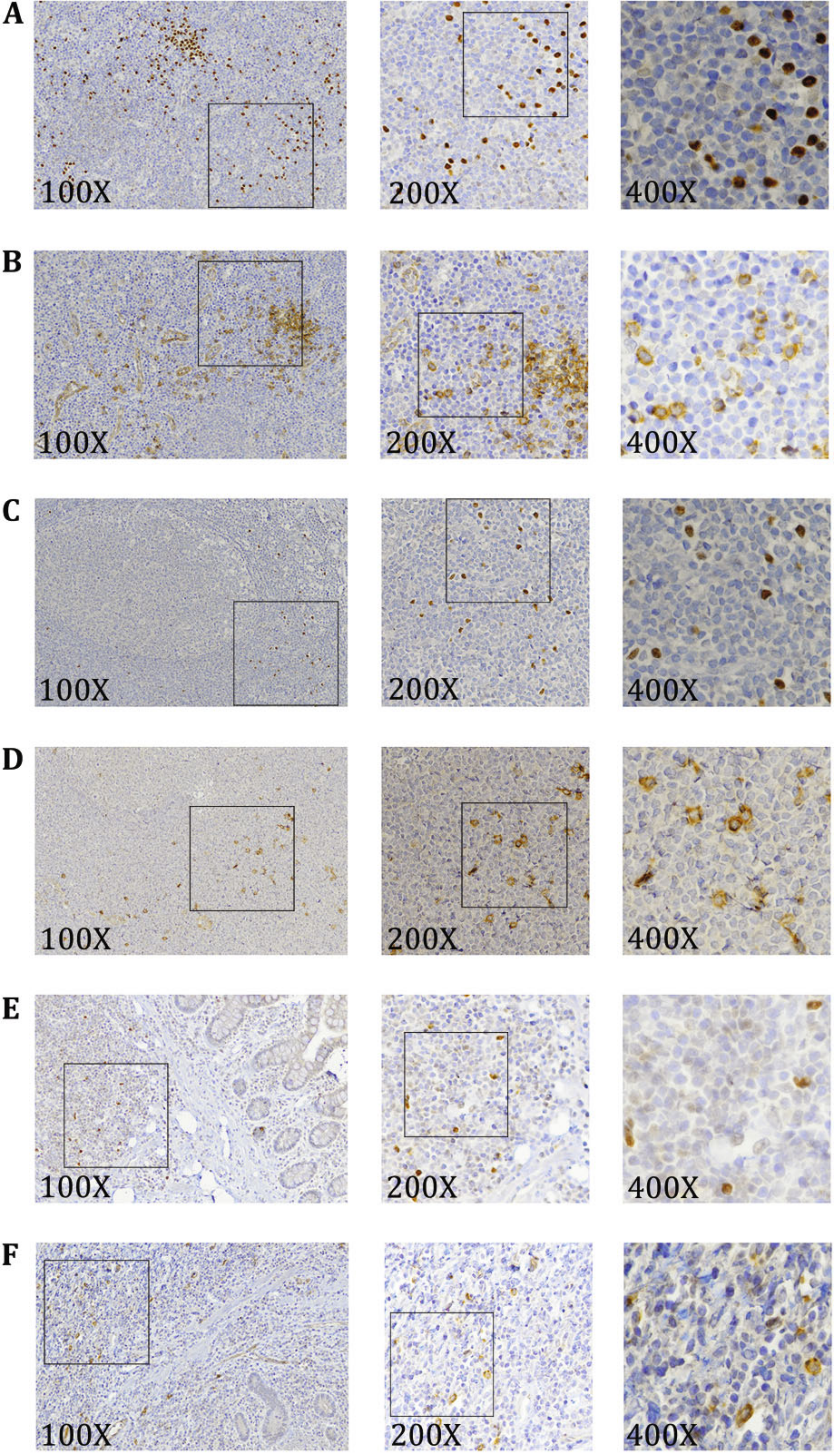
10F7 stains pDCs in different human tissues. The binding specificity of 10F7 (**A**, **C**, and **E**) and anti-CD123 (**B**, **D,** and **F**) antibody was compared on slides with paraffin-embedded human tissue, including breast cancer draining lymph node (**A** and **B**), tonsil (**C** and **D**) and large intestine tissue form colon cancer patient (**E** and **F**) (10F7 and anti-CD123, *brown*; hematine, *blue*)

We tested 10F7 binding of pDCs by immunohistochemical staining in human tissue sections and compared its specificity with the anti-CD123 antibody, which is the most commonly used antibody for pDC staining. As reported previously, we found that the anti-CD123 could stain the cell surface of pDCs. In addition, the high endothelial venules (HEV) are also positive for CD123 staining (Fig. 5B, D). However, 10F7 clearly stained the nuclei of pDCs which agrees with the nuclear localization of E2-2. It is noteworthy that the nuclear staining property of 10F7 distinguished pDCs from other cells around them more clearly.

**Fig. 6:**
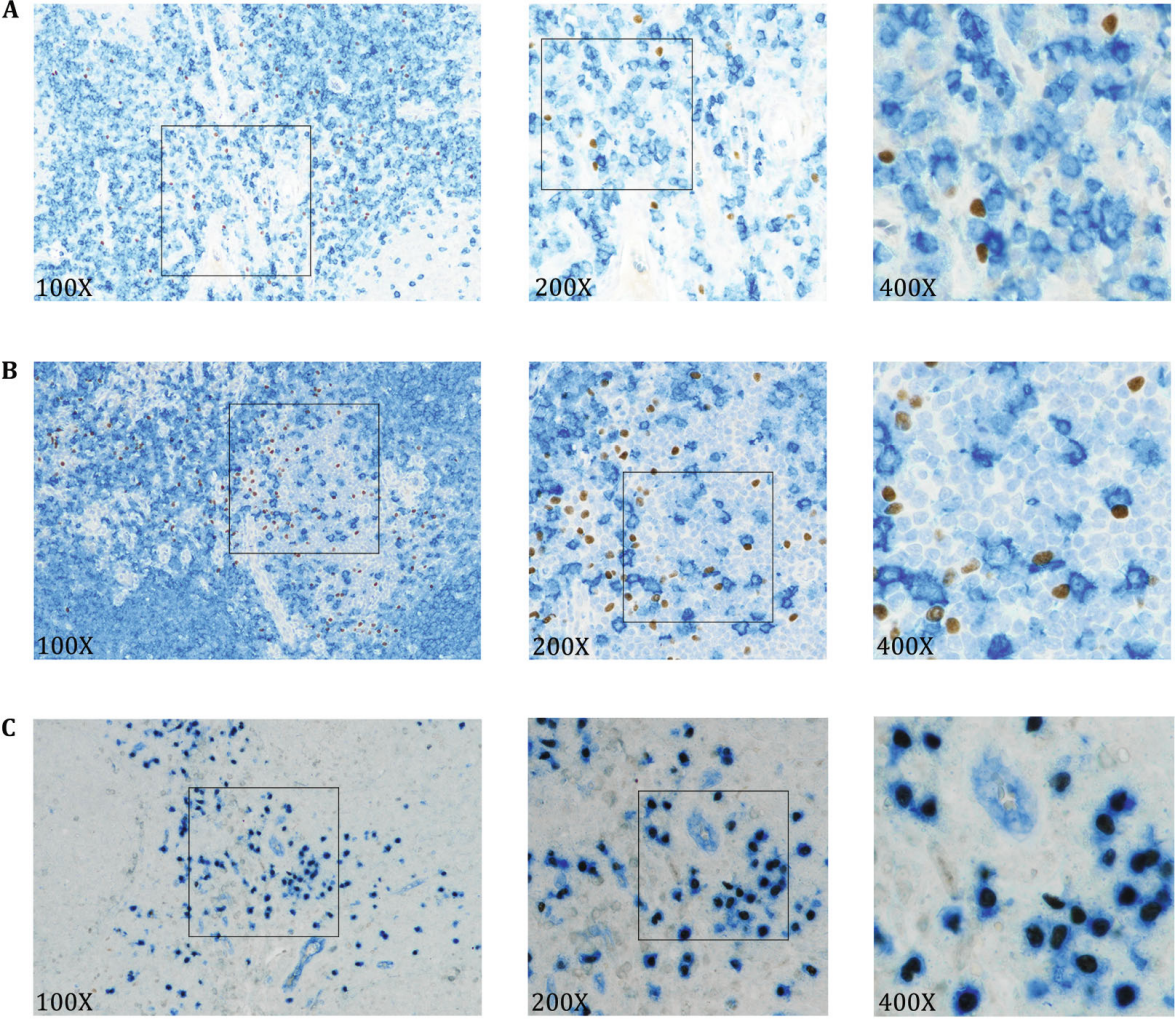
10F7 specifically stains pDCs by immunohistochemistry. The localization of 10F7-stained pDC, the anti-CD3 antibody-stained T cells (**A**) and anti-CD20 antibody-stained B cells (**B**) in human tonsil was analyzed (10F7, *brown*; anti-CD3 and CD20, *dark blue*; hematine, *light blue*). **C** A co-staining of 10F7 and anti-CD123 antibody in human tonsil (10F7, *brown*; anti-CD123, *blue*)

To confirm the binding specificity of 10F7, we also did double-staining of 10F7 with anti-CD3 antibody (T lymphocytes) (Fig. 6A) or anti-CD20 antibody (B lymphocytes) (Fig. 6B). No co-localization of 10F7 with CD3 or CD20 was observed, which confirmed that 10F7 staining is pDC-specific. In addition, double-staining of 10F7 with anti-CD123 antibody (Fig. 6C) also suggests higher specificity of our novel antibody for pDC staining in immunohistochemistry.

### The 10F7 antibody binds to macaque pDCs

**Fig. 7:**
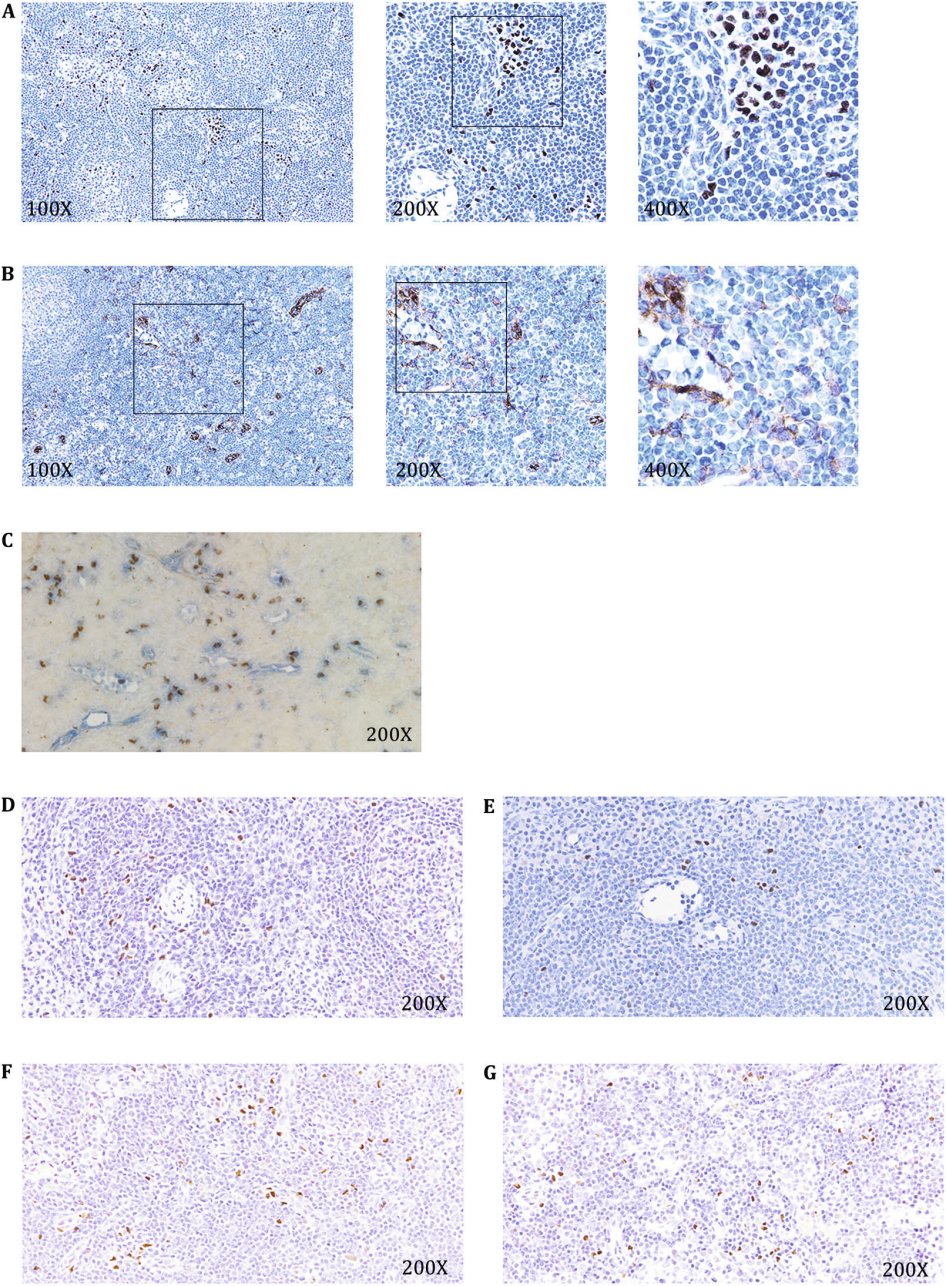
10F7 stains monkey pDCs in the lymph nodes. 10F7 (**A**) and anti-CD123 antibody (**B**) staining of monkey axillary lymph node. (10F7 and anti-CD123 antibody, *brown*; hematine, *blue*). **C** A co-staining of 10F7 and anti-CD123 antibody in monkey sub-maxillary lymph node (10F7, *brown*; anti-CD123 antibody, *blue*). 10F7 staining of monkey spleen (**D**), axillary lymph node (**E**), sub-maxillary lymph node (**F**), and inguinal lymph node (**G**) (10F7, *brown*; hematine, *blue*)

In consistent with the high homology between human and monkey sequences of the selected E2-2 fragment as immunogen, the 10F7 could cross-react with non-human primates (Fig. 7). 10F7 antibody has a similar nuclear staining in the axillary lymph node samples of rhesus macaque (Fig. 7A) and the specificity is higher compared with the anti-CD123 antibody (Fig. 7B, C). To further confirm this in other tissues, we also stained the pDCs with 10F7 in sections of macaque spleen (SP) (Fig. 7D), axillary lymph node (aLN) (Fig. 7E), sub-maxillary lymph node (smLN) (Fig. 7F), and inguinal lymph node (iLN) (Fig. 7G). In summary, 10F7 could stain monkey pDCs specifically on the paraffin-embedded tissue sections.

## DISCUSSION

Although tremendous progresses have been made since the discovery of pDCs (Reizis et al. 2011; Swiecki and Colonna 2015), a specific marker for identifying pDCs in paraffin-embedded tissue sections by immunohistochemistry analysis is still lacking. CD123 has been used as a pDC marker in the immunohistochemistry (Masten et al. 2006; Kutzner et al. 2009; Dave et al. 2012,). However, CD123 also expressed on high endothelial venules (HEVs) or myeloid cells (Vermi et al. 2011). To discriminate pDCs with myeloid dendritic cells (mDCs), Nishikawa used a combination of 2 markers, S100B and fascin, in the immunohistochemistry analysis of human pathological tissues (Nishikawa et al. 2009). In 2013, Montes-Moreno et al. (2013) reported that SPIB might be a novel immunohistochemistry marker for the diagnosis of blastic plasmacytoid dendritic cell neoplasms (BPDCN), one kind of leukemia related to pDCs (Cronin et al. 2012). However, the specificity of this marker is not ideal, as some proportions of the B cell and T-cell lymphomas can also be positive for SPIB (Boiocchi et al. 2013; Montes-Moreno et al. 2013). Boiocchi et al. (2013) proposed that BDCA-2, which is also preferentially expressed in pDCs, should be included as an additional marker to SPIB for the normal or neoplastic pDC staining. In this study, we developed a novel monoclonal antibody, the 10F7, which targets to E2-2, a transcription factor preferentially expressed in pDCs. The immunohistochemistry analysis showed a clear-cut staining of the nucleus of pDCs. Although very low level E2-2 expression in B cells has been documented (Cisse et al. 2008), our study demonstrates no obvious staining in both B and T lymphocytes. In addition, E2-2 is a more specific pDC marker than the commonly used CD123 in the immunohistochemical analysis. Since the amino acid sequence of the truncated E2-2 is conserved among species, 10F7 can also cross-react with pDCs from rhesus macaques, which implied its potent use in the non-human primate model studies. We hope this antibody could serve as an additional tool for immunohistochemistry analysis of pDCs in both clinical and basic research. Additionally, we observed a high homology of E2-2 sequences between different species (Fig. 3). If 10F7 could also stain E2-2 in other species, it will be an ideal tool to study pDC in those animals.

## MATERIALS AND METHODS

### E2-2 protein structure prediction, gene cloning, and expression

The secondary structure of E2-2 was analyzed with online server-Jpred4, and the amino acid sequence from 362 to 671 with three predicted stable alpha-helix domains was selected as immunogen (http://www.compbio.dundee.ac.uk/jpred4/index.html). The coding sequence was cloned into the pET vector with His tag on the C termini and transformed into *E. coli* for the protein production. The protein was purified with the Nickel column and then used as immunogen.

### Animals, immunization, and antibody screening

The BALB/c mice used in this study for the monoclonal antibody preparation were purchased from Vital River and maintained following the national and institutional guidelines for laboratory animals. Mice were immunized subcutaneously once a month for four times with the purified E2-2 fragment at a dosage of 60 μg/mouse with 15 μg CpG1826 as the adjuvant. Three days after the last immunization, the mouse was sacrificed and the spleen was obtained for single cell suspension. At last, the splenocytes were fused with Sp2/0 myeloma cells. The screening was carried out with ELISA.

### Antibody purification

The hybridoma was injected into Rag2^−/−^ γc^−/−^ immunodeficiency mice and ascites was collected. Then the antibody was purified with the protein G Sepharose 4 Fast Flow (GE Healthcare) following the vendor’s instruction. Briefly, the ascites was diluted in the binding buffer (20 mmol/L sodium phosphate, pH 7.0) at a ratio of 1:5. Then the diluted ascites was added to the protein G-Sepharose column. At last, the antibody was collected with the elution buffer (0.1 mol/L glycine, pH 2.5–3.0) and mixed with the neutralizing solution (1 mol/L Tris-HCl, pH 9.0). Then the antibody was washed with PBS (pH 7.0–7.4) and condensed in a 30-kDa Amicon Ultra-15 Centrifugal Filter Units (Amicon).

### RNA extraction, reverse transcription, and Real-time PCR

RNAs of pDCs and PBMCs were extracted from TRIzol reagent (Invitrogen)-treated samples. cDNA was synthesized with Moloney Murine Leukemia Virus Reverse Transcriptase (Promega). The expression level of E2-2 in different samples was tested with CYBR Green mix on the Rotor-Gene Corbett 65HO (Corbett Life Science). The primers used in this study are as follows:

- E2-2 forward 5′-GAGTGTCTCCTCTGGCAGC-3′;
- E2-2 reverse, 5′-CCATGTGATTCGATGCGTC-3′;
- EF-1α forward 5′-ATATGGTTCCTGGCAAGCCC-3′;
- EF-1α reverse, 5′-GTGGG GTGGCAGGTATTAGG-3′.

### Cell sorting with flow cytometry

PBMCs were stained with the following antibodies: mouse anti-human CD3/CD14/CD16/CD19 (lineage) antibody conjugated with FITC, CD11c antibody conjugated with APC, HLA-DR antibody conjugated with APC-Cy7, and CD123 antibody conjugated with BV421. All the antibodies were products of BioLegend. The pDCs were gated as lineage^−^HLA^−^DR^+^CD123^+^ and sorted with the flow cytometer (FACSAria, BD Biosciences).

### Immunohistochemistry

The human and monkey tissues were fixed with 4% paraformaldehyde (PFA) and embedded with paraffin. For antigen retrieval, the EDTA retrieval buffer (pH 9.0) was used. The mouse anti-human CD123 (NCL-L-CD123, Leica Biosystems) was used for comparison in this study. The reagents used for the staining were Polymer HRP Detection System (PV-9002, ZSGB-BIO) for single-staining and DouSP (KIT-9999, MXB) for double-staining. The developing reagents were DAB (ZLI-9018, ZSGB-BIO) and VECTOR BLUE SUBSTRATE KIT (SK-5300, Vector Laboratories, Inc).

Immunohistochemistry images were taken by the Nikon binocular microscope (Eclipse Ci-L, Nikon) and imaging system (DS-Ri2, Nikon).

### Western blotting

The HEK293 T cells were transfected with E2-2 expressing plasmid at different quantities. 48 h later, the cells were harvested and lysed for the preparation of the protein extracts. The extracts were separated with SDS-PAGE and transferred to the polyvinylidene difluoride (PVDF) membrane. The 10F7 antibody, rabbit anti-HA polyclonal antibody, and anti-tubulin were incubated with the membrane overnight at 4 °C. After incubation with the secondary HRP-conjugated antibody, the development was carried out with the HRP substrate (Millipore).

### Statistical analysis

The expression levels of the E2-2 in different cells and tissues were analyzed by GraphPad Prism 6 software. The data shown were means of ±SEM for the result of two samples in BioGPS cDNA array tests and three different samples in our real-time PCR test. Statistically significant differences were determined by unpaired Student’s *t* test, and *P* value < 0.05 was considered as statistically significant.

## Abbreviations

BPDCN: Blastic plasmacytoid dendritic cell neoplasm
ELISA: Enzyme-linked immunosorbent assay
HEV: High endothelial venules
HIV: Human immunodeficiency virus
IHC: Immunohistochemistry
ITF2: Immunoglobulin transcription factor 2
pDC: Plasmacytoid dendritic cell
SEF2: SL3-3 enhancer factor 2
SIV: Simian immunodeficiency virus
TCF4: Transcription factor 4
